# Evaluating the Impact of a National Telehealth Outpatient Mental Health Program in Rural Communities

**DOI:** 10.3390/healthcare14111557

**Published:** 2026-06-02

**Authors:** Melissa M. Matos, Conor O’Neill, Kayla George, Elliot Summers, Erin O’Callaghan

**Affiliations:** 1 Brightside Health, San Francisco, CA 94114, USA; conor.oneill@brightside.com (C.O.);; 2 California State Polytechnic University, Pomona, CA 91768, USA; elliotcsummers@gmail.com

**Keywords:** telemedicine, eHealth, telehealth mental health, rural health, clinical outcomes, patient engagement, access to care

## Abstract

**Highlights:**

**What are the main findings?**

- Rural patients demonstrated comparable engagement and access to care with non-rural patients, with rapid time to first visit (~5 days).
- Both rural and non-rural patients experienced significant and clinically meaningful improvements in depression, anxiety, and suicidal ideation, with approximately 70% achieving MCID and over half reaching remissions, despite rural patients presenting with higher baseline severity and greater medical complexity.

**What are the implications of the main findings?**

- Telehealth mental health services can mitigate geographic disparities and deliver equitable clinical outcomes for rural populations despite greater baseline severity, medical complexity, and socioeconomic challenges.
- Integrated telehealth care models, combining synchronous and asynchronous engagement, access, and meaningful clinical improvement at scale for rural populations, highlighting their value as a viable alternative to in-person care.

**Abstract:**

Background/Objectives: Limited data exist on care delivery, engagement, and clinical outcomes among rural populations accessing telehealth mental health services, particularly within integrated psychotherapy-and-psychiatry models. This retrospective observational 12-week cohort study aimed to examine access, engagement, and preliminary clinical outcomes for rural and non-rural patients receiving services through a national outpatient telehealth mental health program. Engagement and clinical outcomes were expected to be comparable among rural and non-rural patients. Methods: Descriptive and inferential analyses were conducted to examine access to care, engagement, and changes in depressive symptoms, anxiety symptoms, and suicidal ideation among rural and non-rural patients receiving telehealth mental health services over a 12-week treatment period. Clinical outcomes were evaluated using the Patient Health Questionnaire-9 (PHQ-9) and the Generalized Anxiety Disorder-7 (GAD-7). The full sample included 8354 rural and 177,864 non-rural patients and was used to assess access and engagement in treatment. A clinical sample (rural *n* = 2096; non-rural *n* = 43,067) that included patients who completed 12 weeks of care was further examined to assess symptom improvement outcomes. Results: Rural patients demonstrated greater baseline symptom severity and medical complexity relative to non-rural patients. Mean time to first appointment was 5.2 days among rural patients and 5.5 days among non-rural patients, with comparable engagement across groups and rural patients averaging approximately 1–2 touch points per week in care. Patient satisfaction ratings averaged 4.9 out of 5. Within the clinical sample, rural patients demonstrated clinically meaningful symptom improvement across depression and anxiety outcomes. Mean PHQ-9 scores improved by 7.0 points (95% CI: 6.72–7.28), and mean GAD-7 scores improved by 6.1 points (95% CI: 5.83–6.37). Additionally, 70.5% of rural patients achieved a minimal clinically important difference in PHQ-9 or GAD-7 scores, and 66.7% of patients reporting suicidal ideation at baseline no longer endorsed suicidal ideation at endline. Conclusions: These findings support the feasibility and preliminary effectiveness of large-scale virtual mental health care models for rural populations. Rural patients demonstrated engagement and clinical outcomes comparable to non-rural patients despite greater baseline severity and medical complexity.

## 1. Introduction

According to the Health Resources and Services Administration [1], as of 31 March 2025, approximately 122,383,988 people lived in areas designated as mental health professional shortage areas (HPSAs). HPSAs are geographic areas, populations, or facilities, such as state/county hospitals, federally qualified health centers, or correctional facilities, with inadequate access to primary, dental and mental health providers [2]. Rural communities represent some of the geographic areas most vulnerable to these shortages and resultant health disparities. Approximately 23% of individuals living in rural areas have a mental illness, yet more than 75% of the rural counties in the United States are designated HPSAs [3,4]. While research indicates that the prevalence of trauma exposure and most psychiatric disorders is similar among adults in rural and urban settings [5], there are disparities in the outcomes, access and utilization of mental health services. Rural adults receive mental health care less frequently and are more likely to be treated by providers with limited specialized training [6]. Additionally, suicide rates are higher in rural areas, with particularly elevated risk among adults ages 25–34 and older men [7].

A robust body of literature has identified factors that both underlie mental health treatment disparities and place barriers to seeking, accessing, and utilizing mental health services in rural communities. This includes geographic isolation, limited health care facilities and infrastructure, lack of reliable and consistent transportation, shortage of trained and specialty mental health providers, higher rates of poverty, and varied insurance coverage limiting access to care [5,8]. Virtual methods of service delivery are able to mitigate the common access barriers of rural communities and offer feasible and effective treatment that is comparable to in-person care [9,10,11,12]. A systematic review using narrative analysis found that telehealth decreases travel time, improves communication with providers, increases access to care, increases self-awareness, and empowers patients to manage their chronic conditions [13]. In addition, findings show that telehealth services, such as videoconferencing-based telepsychiatry, promote access and high levels of satisfaction among patients [10].

Although the utilization of mental health treatment via telehealth has been extensively studied, there is less research examining the clinical outcomes and engagement patterns specifically among rural populations using large-scale virtual integrated mental health care models. Recently, a national analysis of Medicare mental health specialists found that greater telemedicine uptake was associated with modest increases in care for rural and low-access populations; however, it did not examine engagement or preliminary effectiveness within virtual integrated care settings [14].

The purpose of this study is to examine and compare access, engagement, and clinical outcomes among rural and non-rural patients who accessed services through a national outpatient virtual mental health program during a 12-week treatment period. The significance of this study lies in its contribution to understanding large-scale integrated telehealth mental health care as a feasible and preliminarily effective model among rural populations. To address these aims, a retrospective observational cohort study was conducted using secondary real-world clinical and operational data obtained from a national outpatient virtual mental health program. By examining engagement patterns and clinical outcomes among rural patients compared to non-rural patients, this research provides valuable insight into how integrated telehealth may expand access to large-scale evidence-based care in rural communities. It is expected that rural patients will demonstrate similar engagement patterns and clinical outcomes when compared to non-rural patients.

1. Examine access and engagement patterns among rural patients seeking virtual mental health treatment compared to non-rural patients.
2. Evaluate the preliminary clinical effectiveness of 12 weeks of virtual mental health treatment among rural patients compared to non-rural patients.

## 2. Materials and Methods

### 2.1. Participants

A retrospective observational cohort design was implemented using real-world clinical and operational data collected through a national outpatient virtual mental health program. Two samples were examined in this study: (1) a full sample and (2) a clinical sample. Access and engagement outcomes were examined for the full sample to assess feasibility of care engagement, and clinical outcomes were analyzed for the clinical sample to evaluate symptom improvement after 12 weeks of care.

#### 2.1.1. Full Sample

Participants were eligible for inclusion if they were an adult (18 years old or older), lived in the United States, and enrolled in outpatient mental health services through a Joint Commission-accredited national telehealth company. Participants were included if they received individual psychotherapy and/or psychiatry services between 1 February 2023 and 30 June 2025. Rurality was determined by comparing each patient’s home ZIP code to the USDA Economic Research Service’s (ERS) Rural–Urban Commuting Area (RUCA) codes and dichotomized as rural versus non-rural [15]. RUCA-based rural classification has been widely used in health services and telehealth research to operationalize rurality at the ZIP code level [16]. Patients who endorsed imminent suicide risk or history of psychosis/schizophrenia were excluded from the study. 

#### 2.1.2. Clinical Sample

**Figure 1:**
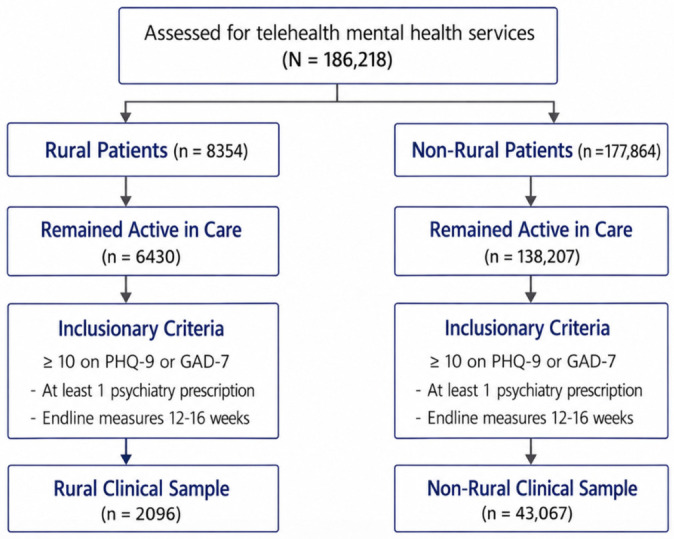
Sample Flow and Clinical Inclusion Criteria for Rural and Non-Rural Patients.

Patients who screened positive for depression or anxiety at baseline, remained active in care at 12 weeks, and provided endline outcome measures between weeks 12 and 16 of treatment were included in the clinical sample. Patients enrolled in psychiatry were included if they completed an initial psychiatric evaluation and received one psychiatric prescription. A positive depression/anxiety screen was defined as a score of ≥10 on the PHQ-9 or GAD-7. Completion of self-report measures (PHQ-9 and GAD-7) at both baseline and endline (collected between weeks 12 and 16) was required for participation. The number of participants assessed for eligibility and included in the clinical sample are presented in Figure 1.

### 2.2. Measures

#### 2.2.1. Depression Symptoms

The Patient Health Questionnaire (PHQ-9) is a 9-item, 4-point Likert scale (0 “not at all” to 3 “nearly everyday”) self-report measure used to screen for and assess depression symptom severity within the past two weeks. Higher scores on the PHQ-9 indicate increased symptom severity and frequency of symptoms. The PHQ-9 has good psychometric properties with 88% sensitivity and 88% specificity [17].

#### 2.2.2. Suicidal Ideation

PHQ-9 item 9 is a specific item related to the frequency of suicidal thoughts (“How often have you been bothered by thoughts that you would be better off dead or of hurting yourself in some way?”). This specific item has been shown to be a predictor of suicide attempts and suicide-related deaths, regardless of age, in medical populations [18].

#### 2.2.3. Anxiety Symptoms

The Generalized Anxiety Disorder-7 (GAD-7) is a 7-item, four-point Likert scale (0 “not at all” to 3 “nearly everyday”) self-report measure used to screen for and assess the severity of Generalized Anxiety Disorder (GAD) symptoms within the past two weeks. Higher scores reflect increased levels of anxiety. The GAD-7 has strong psychometric properties, with 89% sensitivity and 82% specificity for GAD [19,20].

#### 2.2.4. Patient Satisfaction Survey

Patient satisfaction was assessed using a satisfaction survey measuring the extent to which patients were satisfied with their mental health treatment and services. Responses were recorded on a 5-point Likert-style scale using the prompt, “How satisfied were you with your services?” Response options ranged from 1 (very dissatisfied) to 5 (very satisfied) [21]. Analyses used the most recent survey completed within the 12-week treatment period.

### 2.3. Procedures

This study was approved by the WCG Institutional Review Board. All enrolled patients completed consent and an initial digital intake before initiating treatment that included clinically validated measures of depression symptoms (PHQ-9) and anxiety symptoms (GAD-7), along with questions assessing clinical presentation, medical history, and demographic characteristics. Patients self-selected their mental health service plan (combination psychotherapy and psychiatry, psychotherapy only, psychiatry only) and scheduled their initial appointment with their clinician(s) upon enrollment. Psychotherapy services consisted of synchronous individual 45 min sessions delivered via videoconferencing and utilized primarily cognitive behavioral therapy approaches. Psychiatry services consisted of a synchronous psychiatric evaluation followed by recommendations and medical management of symptoms as appropriate. All psychotherapy services were completed by fully licensed mental health therapists (e.g., licensed clinical mental health counselors, licensed clinical social workers, licensed psychologists), and all psychiatry services were provided by a psychiatry provider (e.g., psychiatric nurse practitioners or psychiatrists). After initiating care, patients were prompted to complete periodic self-report measures (i.e., PHQ-9, GAD-7) that provided a measurement-based approach to monitor clinical outcomes. Completion of clinically validated measurement scales was linked to real-time alerts for treating clinicians, such as worsening or high-risk symptoms, including suicidal ideation.

#### 2.3.1. Data Analysis

Patient data were maintained using de-identified databases for analytics and to support detailed examination of patient interactions and treatment outcomes. A 12-week retrospective observational design was used to evaluate patient engagement and clinical outcomes.

##### Access to Care and Patient Engagement

Analyses of access to care and patient engagement were conducted using descriptive methods within the full sample of rural and non-rural patients receiving outpatient telehealth mental health services. Access to care was calculated by the length of time between a patient completing the initial digital intake and attending their first appointment. Appointments were offered within 72 h and patients self-selected their appointment day and time.

The telehealth care model included asynchronous and synchronous touch points between the provider and patient. Patient engagement was measured by calculating an average of these touch points which included: (1) number of psychotherapy sessions attended (for those with a psychotherapy plan), (2) number of psychiatry visits attended (for those with a psychiatry plan), and (3) number of asynchronous messages between the patient and provider(s) and completion of self-report measures (PHQ-9, GAD-7).

##### Clinical Outcomes

Clinical outcomes were evaluated for both the rural and non-rural clinical samples. Within- and between-group (rural vs. non-rural) analyses were conducted comparing average baseline and endline scores (obtained at 12–16 weeks) to evaluate clinical effectiveness and assess rural and non-rural group differences. Treatment programs (e.g., psychiatry, psychotherapy, psychiatry and psychotherapy) were pooled to evaluate broad effectiveness of the comprehensive telehealth model. Paired-sample *t*-tests were used to analyze within-group changes from baseline to endline, and Welch’s independent-sample *t*-test with unequivocal variances was used for between-group comparisons. The effect sizes were calculated using Cohen’s d (mean change divided by the pooled standard deviation of the change in scores). The minimal clinically important differences (MCIDs), defined as “patient derived scores that reflect changes in a clinical intervention that are meaningful for the patient” [22] were examined for both PHQ-9 and GAD-7. Measure-specific MCID for PHQ-9 was defined as a >5-point reduction in scores and GAD-7 as >4-point reduction, consistent with prior research examining MCID in anxiety [23]. Given that patients may present with either or both depressive and anxiety symptoms and could meet clinical thresholds on either measure at baseline, an overall MCID was calculated by aggregating MCID across PHQ-9 and GAD-7. This approach allowed for a comprehensive assessment of clinically meaningful improvement across primary symptom domains. The proportion of patients reaching MCID and time to MCID were evaluated. Remission rates were examined and defined as the proportion of patients achieving a score of <10 score on the PHQ-9 and/or GAD-7 after starting with a corresponding baseline score of 10 or greater. Suicidal ideation elimination rate was defined as the proportion of patients who endorsed suicidal ideation at baseline (PHQ-9 item 9 > 0) and subsequently reported no suicidal ideation (item 9 = 0) after 12 weeks of care.

## 3. Results

### 3.1. Demographics

The full rural sample included 8354 patients (non-rural, *n* = 177,864) enrolled in telehealth outpatient mental health services. Among rural patients, the majority (56.4%) were enrolled in combined psychotherapy and psychiatry services, while 13.8% received psychotherapy only and 29.8% received psychiatry services only. Non-rural patients demonstrated a comparable pattern of service plan enrollment, with 53.6% enrolled in combined treatment, 14.6% in psychotherapy only, and 31.7% in psychiatry only (see Table 1A–C).

The geographic distribution of rural patients was as follows: Southern United States (56.8%, *n* = 1324), Midwest (16.4%, *n* = 369), Northeast (15.9%, *n* = 324), and Western United States (10.9%, *n* = 247). Non-rural patients demonstrated a comparable geographic distribution, with the largest proportion residing in the Southern United States (52.4%, *n* = 93,123), followed by the Western United States (21.4%, *n* = 38,011), and similar proportions in the Midwest (13.4%, *n* = 23,894) and the Northeast (12.8%, *n* = 22,835) (see Table 2).

The majority of rural patients were female (69%), White (86.1%), and between the ages of 25 and 44 years old (25–34 = 33.7%, 35–44 = 26.7%). Among those who reported their socioeconomic status, 32% had an annual income of $60,000 or less ($30,000–$60,000 = 25.7%, <$30,000 = 16.4%), nearly half (46.8%) reported high school as their highest education, and over half were full-time employed (57.2%), with smaller proportion unemployed (29%). Commercial insurance coverage was the most common (52.8%), followed by cash pay plans (24.5%) and commercial exchange (11.7%). Medicare (6%) and Medicaid (4.9%) coverage accounted for 10.9% of the sample. The non-rural sample (*n* = 177,864) was similar in gender (female = 65.4%) and age (25–34 = 37.5%, 35–44 = 25.8%). However, the non-rural sample included a smaller proportion of patients aged 65 years and older compared to the rural sample (2.9% vs. 4.0%, respectively; see Table 1). The non-rural sample was more racially diverse (White = 68.1%) with higher rates of employment (full-time employed = 65.1%) and educational attainment (associate, bachelor’s or advanced degree = 58.2%), with commercial insurance serving as the most common coverage (65.3%), followed by cash pay (22.2%), commercial exchange (6.4%), Medicare (3.8%) and Medicaid (2.3%) coverage (see Table 1).

Major Depressive Disorder was the most common primary mental health diagnosis (rural = 43.8%, non-rural = 43.7%), followed by Generalized Anxiety Disorder (rural = 18.2%, non-rural = 20.4%), and Unspecified Anxiety Disorder (rural = 8.6%, non-rural = 9%). A large proportion of patients presented significant symptoms at baseline, with 82.2% (*n* = 6872) having moderate to severe depression (non-rural = 78.1%) and 80.8% (*n* = 6746) having moderate to severe anxiety (non-rural = 76.9%; see Table 3 and Table 4).

A wide range of comorbid medical conditions was reported among the full sample (see Appendix A—Table A1). Among rural patients, the most frequently reported medical conditions were hypercholesterolemia (26.7%), chronic pain (23.8%), and obesity (22.1%), followed by asthma (10.8%) and thyroid disorders (9.7%). Similar conditions were reported among non-rural patients, with hypercholesterolemia (24.2%), obesity (18.0%), chronic pain (16.9%), and asthma (10.5%) representing the most common conditions.

### 3.2. Access to Care and Patient Engagement

In the full sample, the average length of time between a patient’s enrollment in outpatient mental health services and attending their first appointment was 5.2 days (non-rural = 5.5 days). Across the full sample, rural patients on average demonstrated 19.8 asynchronous and synchronous care touch points (SD = 19.5) during the 12-week treatment period. Non-rural patient care touch points averaged 18 (SD = 18.6) (see Table 5).

### 3.3. Patient Satisfaction

Patient satisfaction with mental health treatment and services was assessed in the full sample. Reviews were submitted by 14.2% of rural patients (*n* = 1188), yielding a mean satisfaction rating of 4.9 out of 5. Similarly, 16.6% of non-rural patients completed satisfaction ratings (*n* = 29,575) with a mean rating of 4.9.

### 3.4. Clinical Outcomes

Across the 12-week treatment period, significant differences were observed between rural (*n* = 2096) and non-rural (*n* = 43, 067) patients at baseline. Rural patients demonstrated slightly higher depressive and anxiety symptom severity (both *p* < 0.001) although suicidal ideation endorsement at baseline did not differ when compared to non-rural patients (*p* = 0.187). As shown in Table 6, co-occurring medical condition count differed significantly between rural and non-rural patients, with rural patients reporting a higher co-occurring medical condition count compared to non-rural patients (M = 1.36 vs. 1.10; *p* < 0.001). These findings indicate that rural patients entered treatment with moderately higher baseline symptom severity and co-occurring medical conditions. Within both rural and non-rural groups, patients entered treatment with an average baseline symptom severity ranging from moderate to severe depression and anxiety, with a large portion endorsing suicidal ideation.

Both rural and non-rural patients demonstrated statistically significant and clinically meaningful improvements in depressive symptoms. Among rural patients, average depressive symptoms, as measured by the PHQ-9, decreased by 41.4% from baseline (M = 16.9) to endline (M = 9.9), reflecting a mean change of −7 points (SD = 6.6; d = −1.06; *p* < 0.001) (see Table 7). Similar improvements were observed among non-rural patients (*n* = 43,067), who demonstrated a 43% reduction in depressive symptoms from baseline (M = 16.5) to endline (M = 9.4), with a mean change of −7.1 points (SD = 6.4; d = −1.10; *p* < 0.001). With regards to depressive symptoms, 62.5% of rural patients achieved MCID, within a mean time of 80 days. Remission was achieved by 50.7% of rural patients at endline. In non-rural patients, 62.5% achieved MCID for depression, with a mean time to MCID at 83.3 days, and 52.6% achieved remission. As shown in Table 6, differences in depressive symptoms between rural and non-rural patients were not statistically significant (*p* = 0.592).

Anxiety symptoms significantly declined over the treatment period across both groups. Among rural patients, average GAD-7 scores decreased by 39.2% from baseline (M = 15.4) to the last assessment (M = 9.3), reflecting a mean change of −6.0 points (SD = 6.2; d = −0.97; *p* < 0.001). Among rural patients, 61.3% achieved MCID for anxiety, with a mean time to MCID of 80.1 days, and 51.7% met remission criteria at endline. Non-rural patients demonstrated a similar pattern, with a 42.4% reduction in anxiety symptoms from baseline (M = 15) to endline (M = 8.6), reflecting a mean change of −6.4 points (SD = 6.1; d = −1.05; *p* < 0.001). Among non-rural patients, 63.3% achieved MCID for anxiety, with a mean time to MCID of 83.1 days, and 56.4% met remission criteria. Although improvements in anxiety symptoms differed significantly between rural and non-rural patients (*p* = 0.02), the effect size was minimal (d = 0.05).

The composite MCID was used to determine the total percentage of patients who achieved MCID in either depression or anxiety. Among rural patients, 70.5% reached MCID on at least one measure (PHQ-9 or GAD-7) and the average time to reach MCID on at least one measure was 74.2 days. Among non-rural patients, 70.9% reached MCID on either the PHQ-9 or GAD-7, and the average time to MCID across measures was 75.4 days. There was no difference in time to composite MCID between rural and non-rural patients (*p* = 0.55).

**Figure 2:**
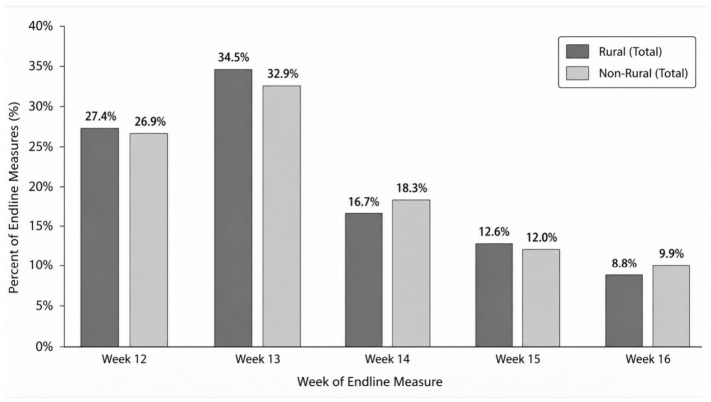
Distribution of the Endline Measures by Assessment Timeframe in the Clinical Sample.

Meaningful reductions were also found among patients endorsing suicidal ideation at baseline. In the rural clinical sample, 41.0% of patients endorsed suicidal ideation at baseline, and 66.7% of those individuals achieved elimination of suicidal ideation by week 12. A similar pattern was observed among non-rural patients, as 39.8% reported suicidal ideation at baseline, and 68.9% achieved elimination by week 12. There were no significant differences in suicidal ideation elimination between rural and non-rural patients (*p* = 0.625). The distribution of the timeframe for completed endline measures across the clinical sample is listed in Figure 2.

## 4. Discussion

The present study examined access, patient engagement and clinical outcomes to investigate the feasibility and preliminary effectiveness associated with a comprehensive telehealth model to address the mental health needs of rural patients at scale in the United States. A full sample was used to analyze access, engagement patterns, demographics, geographic distribution, primary mental health diagnoses, and baseline symptom severity among adults receiving outpatient mental health services through a national, Joint Commission-accredited telehealth organization. A clinical subsample was examined to more precisely evaluate observed clinical outcomes and included patients who were actively receiving mental health treatment and had complete outcome data. Using both samples allowed the study to place clinical outcomes within the context of the broader care population and evaluate both feasibility at scale as well as preliminary clinical effectiveness. A non-rural sample was used to compare and contextualize the results of the rural samples.

The full rural sample largely resided in the Southern U.S. (56.8%) and was predominantly White (86%), female (69%) and between the ages of 25 and 44 (60%). Over half of the sample achieved a high school diploma or less, and nearly a third were unemployed. Patients presented primarily with moderate to severe baseline depressive and anxiety symptoms, and over 40% endorsed suicidal ideation at the start of care.

The non-rural sample was demographically similar to the rural sample with a few key differences. Non-rural patients demonstrated greater racial diversity, higher rates of full-time employment (65.1%), and higher levels of educational attainment (associate’s, bachelor’s, or advanced degrees; 58.2%). Commercial insurance was the most common form of coverage among both rural and non-rural participants, though the non-rural sample had a notably lower rate of Medicare and Medicaid coverage (6%) compared to the rural group (10.9%). The majority of patients in both rural and non-rural groups reported at least one co-occurring medical condition (see Appendix A—Table A2); however, rural patients demonstrated higher rates of multiple medical conditions, with 31.6% reporting two or more diagnoses.

Engagement and access were comparable across groups. Rural and non-rural patients were typically seen by a provider in approximately five days, indicating rapid access to mental health care through telehealth. Importantly, patients chose their initial appointment day and time, and earlier sessions may have been available consistent with the organization’s standard of offering appointments within 24–72 h. In comparison, the national average wait time for behavioral health services across the U.S. was 48 days [24], with one study on psychiatry appointment availability reporting median wait times for in-person services at 67 days and telepsychiatry appointments at 43 days [25]. Providing fast access to treatment via telehealth may help address known care barriers including provider shortages and extended wait times in rural areas.

Once enrolled in care, patients engaged with their providers in two ways: asynchronous care touch points that included messaging and self-report symptom measure assessments, and live synchronous video sessions. Together, these touch points were designed to support an overall care experience that allowed for multiple methods of engagement, follow through, and iterations of care. Real-time alerts from asynchronous touch points were surfaced to the provider allowing for immediate action and support via messaging, as well as prompts to schedule and complete synchronous sessions. Patients averaged nearly one to two touch points per week, demonstrating high rates of engagement in the care process. These findings suggest that telehealth models combining synchronous and asynchronous experiences may offer a unique and feasible opportunity to engage with patients in rural settings who may have difficulties accessing treatment otherwise restricted to in-person treatment.

Among patients who engaged in treatment and completed follow-up assessments, rural and non-rural patients demonstrated clinically meaningful improvements in depression, anxiety, and suicidal ideation, with nearly three-quarters of patients having achieved an MCID and over half having met remission criteria. The majority of patients reporting suicidal ideation at baseline experienced meaningful reductions in severity, and nearly two-thirds of patients who reported suicidal ideation at baseline reported no suicidal ideation by their last self-reported assessment. While the observational study design limits inferential power, these results offer preliminary evidence that integrated behavioral telehealth utilizing asynchronous and synchronous care models may yield meaningful clinical improvement in rural patients after 12 weeks of treatment.

Several limitations should be considered when interpreting these findings. Suicidal ideation outcomes were assessed using PHQ-9 item 9, which has been validated as a screening measure for suicidal ideation; however, it is not equivalent to a comprehensive suicide risk assessment and may not fully capture the complexity of suicidality. Additionally, clinical outcomes were evaluated among patients who remained engaged in care and completed endline outcome assessments, which may introduce potential selection and attrition bias. Characteristics of patients who completed follow-up assessments may differ from those who discontinued care or did not complete endline measures. In addition, endline outcome assessments were collected across a 12–16-week interval, introducing some variability in follow-up duration that may influence interpretation of symptom change over time. The imbalance in sample size between rural and non-rural groups may also contribute to statistically significant findings despite relatively small effect sizes across some comparisons. Nevertheless, both rural and non-rural patients demonstrated clinically meaningful improvements in depressive and anxiety symptoms over the treatment period.

Importantly, rural patients demonstrated clinical outcomes comparable to those of non-rural patients despite moderately higher baseline symptom severity, increased co-morbid medical complexities, and lower rates of education and employment. While encouraging, the real-world observational data were unadjusted, introducing the potential for confounding variable effects that should be considered with interpretation. These initial findings may be strengthened with future studies comparing in-person to virtual care options. For example, there is limited quasi-experimental evidence that telehealth conditions yield improvements in depression and anxiety scores comparable to in-person care overall [26]. Still, randomized controlled trials comparing virtual care to in-person care specifically with rural populations are needed to further establish the unique benefits of virtual care models in improving access, engagement, and clinical effectiveness. In addition, the present study examined clinical outcomes collectively, which included patients receiving psychotherapy only, psychiatry only, or both services. While this approach and the corresponding results support the feasibility and preliminary effectiveness of a comprehensive treatment model, it limits interpretation regarding modality-specific outcomes and the relative contribution of psychotherapy, psychiatry, or integrated care services. Additional research designed to examine the distinct impact of each service would be beneficial.

A considerable proportion of the present rural sample faced socioeconomic and health-related challenges (e.g., 29% unemployed, 42.1% annual income <$60K, a third reporting two or more co-occurring medical conditions), which are known to influence mental health outcomes. Future studies will benefit from analyzing the unique impact that social determinants of health may have on rural patient care administered virtually.

The generalizability of these findings is potentially limited by the rural sample size and demographics. According to the 2020 U.S. Census, approximately 20% of the United States population lives in rural areas [27]. In comparison, approximately 5% of the full sample (*N* = 186,218) was identified as living in rural communities, suggesting an underrepresentation of rural individuals in the current study sample when compared to the national population. Inference of these findings may be appropriately generalized to the proportion of rural individuals with moderate to severe mental health symptoms that are actively seeking mental health treatment, which may differ from broader population estimates. Policy and health care initiatives should continue to focus on increasing access to rural populations, especially given that they may present with higher rates of severity and complexity and lack effective and timely treatment options.

## 5. Conclusions

Mental health care is underutilized by rural communities due to limited availability and access to high-quality, effective treatments that have historically been limited to in-person care options. Over the past several years, evidence has continued to show that telehealth treatment models offer solutions to known access barriers without sacrificing clinical quality and effectiveness. These findings support the feasibility and preliminary effectiveness of telehealth-based mental health services delivered at scale for rural populations. Additional controlled and longitudinal research is recommended to further evaluate comparative effectiveness, long-term outcomes, and mechanisms influencing engagement and symptom improvement.

## Figures and Tables

**Table 1:** (**A**) Full Sample Demographics by Rurality. (**B**) Full Sample Sociodemographics by Rurality. (**C**) Full Sample Insurance and Service Plan by Rurality.

(**A**)		
**Demographic**	**Rural (*n* = 8354), *n* (%)**	**Non-Rural (*n* = 177,864), *n* (%)**
**Gender**		
Female	5795 (69.4%)	116,327 (65.4%)
Male	2416 (28.9%)	58,554 (32.9%)
Not Available	143 (1.7%)	2983 (1.7%)
**Gender Identity**		
Female	5766 (69.0%)	114,845 (64.6%)
Male	2403 (28.8%)	58,191 (32.7%)
Gender variant	100 (1.2%)	2978 (1.7%)
Transgender female	23 (0.3%)	401 (0.2%)
Transgender male	33 (0.4%)	725 (0.4%)
Not sure	17 (0.2%)	566 (0.3%)
Not Available	12 (0.1%)	158 (0.1%)
**Age**		
18–24	1185 (14.2%)	28,566 (16.1%)
25–34	2814 (33.7%)	66,772 (37.5%)
35–44	2231 (26.7%)	45,913 (25.8%)
45–54	1222 (14.6%)	21,784 (12.2%)
55–64	568 (6.8%)	9686 (5.4%)
≥65	334 (4.0%)	5143 (2.9%)
**Race/Ethnicity**		
White	7190 (86.1%)	121,068 (68.1%)
Black	388 (4.6%)	17,592 (9.9%)
Hispanic	352 (4.2%)	20,221 (11.4%)
Asian	51 (0.6%)	7019 (3.9%)
Native American	79 (0.9%)	968 (0.5%)
Pacific Islander	8 (0.1%)	461 (0.3%)
Other	227 (2.7%)	9027 (5.1%)
Not Available	59 (0.7%)	1508 (0.8%)
(**B**)		
**Sociodemographic**	**Rural (*n* = 8354), *n* (%)**	**Non-Rural (*n* = 177,864), *n* (%)**
**Income**		
<$30,000	1373 (16.4%)	23,069 (13.0%)
$30,000–$60,000	2144 (25.7%)	44,393 (25.0%)
$60,000–$100,000	1233 (14.8%)	33,752 (19.0%)
>$100,000	704 (8.4%)	26,917 (15.1%)
Not Available	2900 (34.7%)	50,733 (28.5%)
**Education**		
High school or less	4430 (53.1%)	72,102 (40.6%)
Associate degree	1436 (17.2%)	25,590 (14.4%)
Bachelor’s degree	1555 (18.6%)	49,744 (28.0%)
Advanced degree	792 (9.5%)	28,087 (15.8%)
Not Available	141 (1.7%)	2341 (1.3%)
**Employment**		
Full time	4779 (57.2%)	115,809 (65.1%)
Part time	832 (10.0%)	17,242 (9.7%)
Unemployed	2421 (29.0%)	38,450 (21.6%)
Not Available	322 (3.9%)	6363 (3.6%)
(**C**)		
**Characteristic**	**Rural (*n* = 8354), *n* (%)**	**Non-Rural (*n* = 177,864), *n* (%)**
**Insurance**		
Cash	2044 (24.5%)	39,434 (22.2%)
Commercial	4442 (52.9%)	116,315 (65.4%)
Commercial Exchange	981 (11.7%)	11,357 (6.4%)
Medicaid	408 (4.9%)	3995 (2.2%)
Medicare	499 (6.0%)	6763 (3.8%)
**Service Plan**		
Psychotherapy	1154 (13.8%)	26,057 (14.6%)
Psychiatry	2487 (29.8%)	56,428 (31.7%)
Psychotherapy and Psychiatry	4713 (56.4%)	95,379 (53.6%)

Note: values are presented as *n* (%).

**Table 2:** Geographic Distribution by Rurality (Full Sample).

U.S. Region Distribution		
Region	Rural (*n* = 8354), *n* (%)	Non-Rural (*n* = 177,864), *n* (%)
Midwest	1369 (16.4%)	23,894 (13.4%)
Northeast	1324 (15.9%)	22,835 (12.8%)
South	4747 (56.8%)	93,123 (52.4%)
West	914 (10.9%)	38,011 (21.4%)

Note: values are presented as *n* (%).

**Table 3:** Primary Mental Health Diagnoses by Rurality (Full Sample).

Primary Diagnosis	Rural (*n* = 8354), *n* (%)	Non-Rural (*n* = 177,864), *n* (%)
Major Depressive Disorder	3658 (43.8%)	77,758 (43.7%)
Generalized Anxiety Disorder	1521 (18.2%)	36,218 (20.4%)
Anxiety Disorder, Unspecified	720 (8.6%)	15,950 (9.0%)
Bipolar II Disorder	484 (5.8%)	8399 (4.7%)
Adjustment Disorder	376 (4.5%)	9600 (5.4%)
Post-Traumatic Stress Disorder	266 (3.2%)	3781 (2.1%)
Bipolar Disorder (Unspecified)	239 (2.9%)	3057 (1.7%)
Bipolar I Disorder	238 (2.8%)	3326 (1.9%)
Persistent Depressive Disorder	233 (2.8%)	4294 (2.4%)
Attention-Deficit/Hyperactivity Disorder	174 (2.1%)	4408 (2.5%)
Panic Disorder	76 (0.9%)	1996 (1.1%)
Obsessive-Compulsive Disorder	79 (0.9%)	1916 (1.1%)
Social Anxiety Disorder	57 (0.7%)	1479 (0.8%)
Borderline Personality Disorder	57 (0.7%)	888 (0.5%)
Postpartum Depression	33 (0.4%)	1099 (0.6%)
Insomnia	29 (0.3%)	758 (0.4%)
Acute Stress Disorder	33 (0.4%)	727 (0.4%)
Alcohol Use Disorder	23 (0.3%)	570 (0.3%)

Note: Values are presented as *n* (%). Diagnoses representing <0.1% of the sample are omitted for clarity.

**Table 4:** Baseline Depression (PHQ-9) and Anxiety (GAD-7) Severity by Rurality (Full Sample).

PHQ-9 Baseline Severity		
Severity Level	Rural (*n* = 8354), *n* (%)	Non-Rural (*n* = 177,864), *n* (%)
Minimal	406 (4.9%)	10,956 (6.2%)
Mild	1076 (12.9%)	28,197 (15.9%)
Moderate	1863 (22.3%)	42,069 (23.7%)
Moderately severe	2416 (28.9%)	48,697 (27.4%)
Severe	2593 (31.0%)	>47,945 (27.0%)
**GAD-7 Baseline Severity**		
**Severity Level**	**Rural (*n* = 8354), *n* (%)**	**Non-Rural (*n* = 177,864), *n* (%)**
Minimal	466 (5.6%)	11,308 (6.4%)
Mild	1142 (13.7%)	29,817 (16.8%)
Moderate	2004 (24.0%)	45,629 (25.7%)
Severe	4742 (56.8%)	91,110 (51.2%)

Note: Values are presented as *n* (%). Severity categories reflect standard PHQ-9 and GAD-7 cutoffs.

**Table 5:** Care Engagement and Access by Rurality (Full Sample).

Metric	Rural (*n* = 8354), M (SD)	Non-Rural (*n* = 177,864), M (SD)
Care touch points	19.8 (19.5)	18 (18.6)
Days to first booking	5.2	5.5

Note: Values represent mean time (in days) from enrollment to the first attended appointment and mean number of care touch points with standard deviations in parentheses.

**Table 6:** Between-Group Comparisons of Clinical Outcomes at 12 Weeks by Rurality.

Metric	Rural (*n* = 2096)	95% CI	Non-Rural (*n* = 43,067)	95% CI	*p*	d
**PHQ-9**						
Baseline, M (SD)	16.93 (5.34)	16.70–17.16	16.50 (5.31)	16.45–16.55	<0.001	0.08
Endline, M (SD)	9.93 (6.55)	9.65–10.21	9.41 (6.27)	9.35–9.47	<0.001	0.08
Mean Change, M (SD)	−7.00 (6.57)	−7.28–−6.72	−7.08 (6.42)	−7.14–−7.02	0.592	0.01
Percent Change (%)	−41.4%	—	−43.0%	—	—	—
MCID Achieved, %	62.5%	—	62.5%	—	—	—
Remission, %	50.7%	—	52.6%	—	—	—
**GAD-7**						
Baseline, M (SD)	15.44 (4.34)	15.25–15.63	15.02 (4.38)	14.98–15.06	<0.001	0.10
Endline, M (SD)	9.39 (6.20)	9.12–9.66	8.65 (5.95)	8.59–8.71	<0.001	0.12
Mean Change, M (SD)	−6.04 (6.20)	−6.31–−5.77	−6.36 (6.06)	−6.42–−6.30	0.023	0.05
Percent Change (%)	−39.2%	—	−42.4%	—	—	—
MCID Achieved, %	61.3%	—	63.3%	—	—	—
Remission, %	51.7%	—	56.4%	—	—	—
**Suicidal Ideation**						
Baseline, M (SD)	0.7 (0.9)	0.66–0.74	0.6 (0.9)	0.59–0.61	0.187	0.03
Mean Change, M (SD)	−0.5 (0.9)	−0.54–−0.46	−0.4 (0.9)	−0.41–−0.39	0.625	0.01
Baseline Endorsement, %	41.0%	—	39.8%	—	—	—
Elimination at 12 Weeks, %	66.7%	—	68.9%	—	—	—
**Medical Conditions**						
Medical Condition Count, M (SD)	1.36 (1.65)	1.29–1.43	1.10 (1.42)	1.09–1.11	<0.001	0.18

Note: CI = 95% confidence interval; M = mean; SD = standard deviation; d = Cohen’s d for within-group effect size, calculated using the mean change score divided by the standard deviation of the change scores. Values are presented as means (M) or percentages, as indicated. Endline refers to the final assessment within the 12-week treatment period. MCID = minimal clinically important difference, defined as a ≥5-point reduction on the PHQ-9 or a ≥4-point reduction on the GAD-7. SI = suicidal ideation measured using PHQ-9 item 9.

**Table 7:** Within-Group Changes in Clinical Outcomes at 12 Weeks by Rurality.

Group	Baseline M (SD)	Endline M (SD)	Mean Change (SD)	95% CI	d	*p*
**Depressive Symptoms (PHQ-9)**						
Rural	16.9 (5.3)	9.9 (6.6)	7.0 (6.6)	6.72–7.28	−1.06	<0.001
Non-rural	16.5 (5.3)	9.4 (6.3)	7.1 (6.4)	7.04–7.16	−1.10	<0.001
**Anxiety Symptoms (GAD-7)**						
Rural	15.4 (4.3)	9.3 (6.2)	6.1 (6.2)	5.83–6.37	−0.97	<0.001
Non-rural	15.0 (4.4)	8.6 (6.0)	6.4 (6.1)	6.34–6.46	−1.05	<0.001

Note: M = mean; SD = standard deviation; mean change = baseline score − endline score; CI = 95% confidence interval for the mean change score; d = Cohen’s d for within-group effect size, calculated using the mean change score divided by the standard deviation of the change scores; endline refers to the final assessment within the 12-week treatment period.

## Data Availability

Restrictions apply to the availability of these data. The data were obtained from a proprietary clinical database and are not publicly available due to privacy and legal restrictions. Data may be available from the corresponding author upon reasonable request and with the permission of the data provider.

## Appendix A

**Table A1:** Self-Reported Medical Conditions by Rurality (Full Sample).

Medical Condition	Rural *n* (%) (*N* = 8354)	Non-Rural *n* (%) (*N* = 177,864)
Arrhythmia	207 (2.5%)	3329 (1.9%)
Asthma	905 (10.8%)	18,731 (10.5%)
Cancer	98 (1.2%)	1493 (0.8%)
Hypercholesterolemia	2229 (26.7%)	43,008 (24.2%)
Chronic pain	1986 (23.8%)	30,009 (16.9%)
Diabetes	463 (5.5%)	7594 (4.3%)
Eating disorder	264 (3.2%)	5938 (3.3%)
Fibromyalgia	428 (5.1%)	5238 (2.9%)
Heart condition	163 (2.0%)	2510 (1.4%)
IBD/Crohn’s	316 (3.8%)	5800 (3.3%)
Kidney	45 (0.5%)	701 (0.4%)
Liver	47 (0.6%)	777 (0.4%)
Long QT	5 (0.1%)	81 (0.0%)
Lung condition	77 (0.9%)	1061 (0.6%)
Obesity	1847 (22.1%)	31,998 (18.0%)
Seizures	28 (0.3%)	689 (0.4%)
Thyroid	812 (9.7%)	12,810 (7.2%)

Note: Values are presented as *n* (%). Percentages are calculated within the rurality category. HLD = hypercholesterolemia. Medical conditions are self-reported.

**Table A2:** Self-Reported Chronic Condition Count by Rurality (Full Sample).

Chronic Condition Count	Rural *n* (%) (*N* = 8354)	Non-Rural *n* (%) (*N* = 177,860)
Not Available	13 (0.2%)	194 (0.1%)
0	3360 (40.2%)	83,026 (46.7%)
1	2336 (28.0%)	50,039 (28.1%)
2	1286 (15.4%)	24,284 (13.7%)
3	576 (6.9%)	10,201 (5.7%)
4	337 (4.0%)	4780 (2.7%)
5	196 (2.3%)	2568 (1.4%)
6	128 (1.5%)	1429 (0.8%)
7	84 (1.0%)	776 (0.4%)
8	22 (0.3%)	371 (0.2%)
9	8 (0.1%)	127 (0.1%)
10	5 (0.1%)	43 (0.0%)
11	3 (0.0%)	17 (0.0%)
12	—	4 (0.0%)
13	—	1 (0.0%)

Note: Values are presented as *n* (%). Percentages are calculated within the rurality category. The chronic condition count reflects the number of self-reported chronic medical conditions.
